# Dichlorophenylpyridine-Based Molecules Inhibit Furin through an Induced-Fit Mechanism

**DOI:** 10.1021/acschembio.2c00103

**Published:** 2022-04-04

**Authors:** Sven O. Dahms, Gisela Schnapp, Martin Winter, Frank H. Büttner, Marco Schlepütz, Christian Gnamm, Alexander Pautsch, Hans Brandstetter

**Affiliations:** † Department of Biosciences and Medical Biology, University of Salzburg, Hellbrunner Straße 34, A-5020 Salzburg, Austria; ‡ Department of Medicinal Chemistry, Boehringer Ingelheim Pharma GmbH& Co KG, Birkendorfer Straße 65, 88397 Biberach an der Riß, Germany; § Department of Drug Discovery Sciences, Boehringer Ingelheim Pharma GmbH& Co KG, Birkendorfer Straße 65, 88397 Biberach an der Riß, Germany; ∥ Department of I&R Research, R&D Project Management and Development Strategies, Boehringer Ingelheim Pharma GmbH& Co KG, Birkendorfer Straße 65, 88397 Biberach an der Riß, Germany

## Abstract

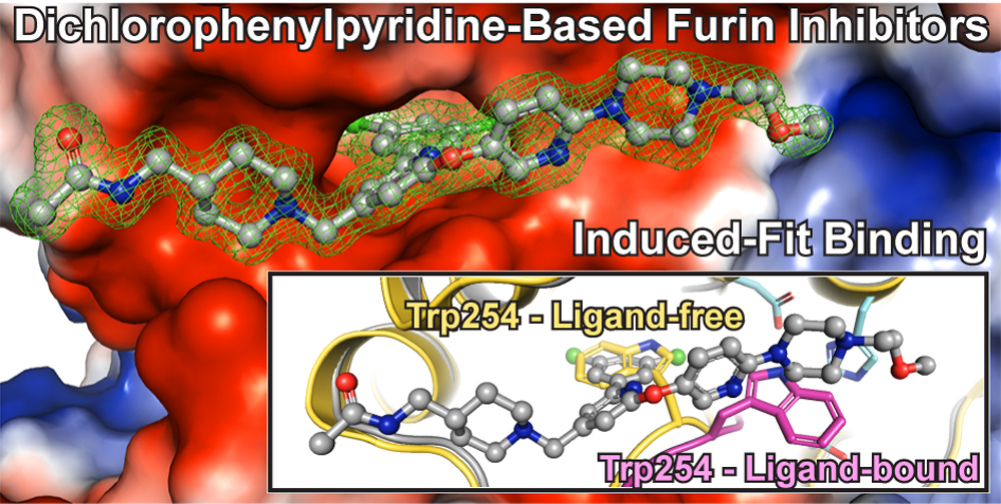

Inhibitors of the proprotein convertase furin might serve as broad-spectrum antiviral therapeutics. High cellular potency and antiviral activity against acute respiratory syndrome coronavirus 2 (SARS-CoV-2) have been reported for (3,5-dichlorophenyl)pyridine-derived furin inhibitors. Here we characterized the binding mechanism of this inhibitor class using structural, biophysical, and biochemical methods. We established a MALDI-TOF-MS-based furin activity assay, determined IC_50_ values, and solved X-ray structures of (3,5-dichlorophenyl)pyridine-derived compounds in complex with furin. The inhibitors induced a substantial conformational rearrangement of the active-site cleft by exposing a central buried tryptophan residue. These changes formed an extended hydrophobic surface patch where the 3,5-dichlorophenyl moiety of the inhibitors was inserted into a newly formed binding pocket. Consistent with these structural rearrangements, we observed slow off-rate binding kinetics and strong structural stabilization in surface plasmon resonance and differential scanning fluorimetry experiments, respectively. The discovered furin conformation offers new opportunities for structure-based drug discovery.

## Introduction

Furin is one of the proprotein convertases (PCs), a family of subtilisin-like proteases involved in the maturation of many secreted proteins. PCs are Ca^2+^-dependent serine endoproteinases harboring a catalytic domain with structural homology to subtilisin.^1^ The so-called kexin/furin-like mammalian PC family members (furin, PC1, PC2, PC4, PACE4, PC5/6, and PC7) recognize multibasic substrate sequences and cleave after the common pattern (R/K)X_*n*_(R)↓ (where *n* = 0, 2, 4, 6; X represents any amino acid; and “↓” marks the scissile peptide bond).^1,2^ Furin, often regarded as the prototypical PC, is the best-characterized member of this protease family and prefers the consensus cleavage motif R-X-K/R-R↓.^3−5^

Unbalanced activity of furin and other PCs is connected to several pathologies such as rheumatoid arthritis, obesity, and cancer as well as infections by bacteria and viruses.^1,2^ Many viral glycoproteins require cleavage by furin, including the S protein of severe acute respiratory syndrome virus 2 (SARS-CoV-2).^6−8^ Several studies have shown that furin inhibitors are efficient suppressors of viral replication.^9^ Acquisition of a furin cleavage site is regarded as a major pathogenicity factor of viruses. Thus, furin inhibitors might serve as broad-spectrum antiviral therapeutics that are also capable for treatment of newly emerging viruses or virus variants.

The multibasic consensus cleavage sequence of furin has been utilized to develop very potent substrate-like inhibitors reaching *K*_i_ values down to the low-picomolar range (see, e.g., ref (10)). Such compounds usually include a number of positively charged amino acids that limit the bioavailability and can result in toxicity in mice.^11^ Nonetheless, substrate-like inhibitors were significantly improved by substitution of arginine with less basic canavanine.^11^ Canavanine-based inhibitors showed a strong antiviral effect in cells at 0.5 μM and reduced toxicity.^7,11^

Beyond substrate-like inhibitors, several types of small-molecule furin inhibitors have been described, including 2,5-dideoxystreptamine- and guanylhydrazone-derived compounds.^12,13^ Structural studies revealed different interaction patterns of these compound classes compared with canonical furin inhibitors.^14,15^ Thus, noncanonical small-molecule furin inhibitors might be a promising opportunity to identify more drug-like compounds with improved bioavailability.

**Figure 1:**
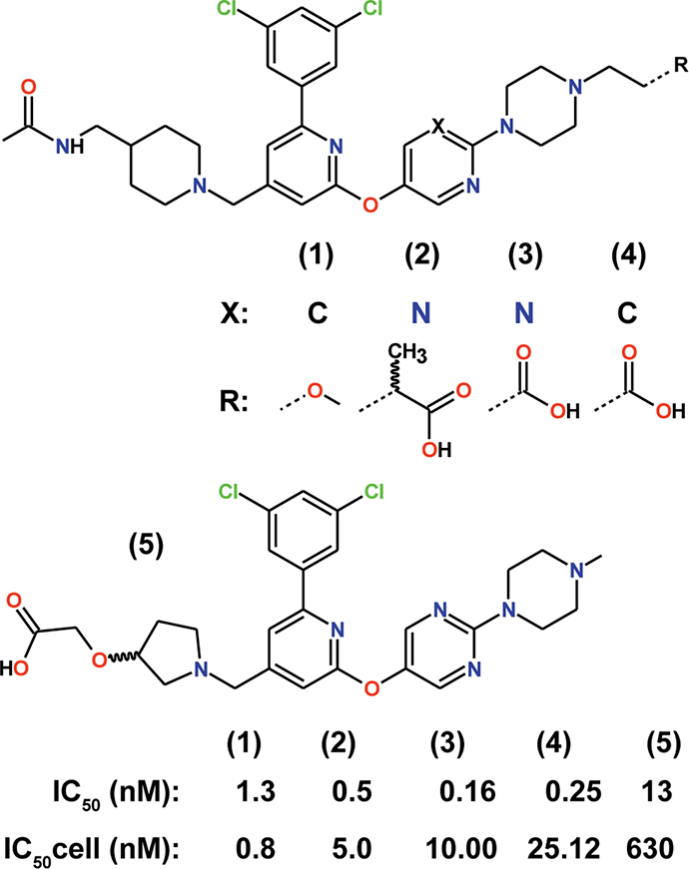
Overview of the inhibitors used in this study. IC_50_ values from enzyme kinetics measurements (IC_50_) and from cell-based inhibition assays (IC_50_cell) are given as reported in the literature.^16^

Recently, a novel class of furin inhibitors containing a (3,5-dichlorophenyl)pyridine core motif were disclosed in a patent by GlaxoSmithKline.^16^ The most intriguing features of these inhibitors are the comparable high potency, which reached 0.8 nM in cell-based assays (IC_50_cell for inhibitor **1**; Figure 1),^16^ and their high efficacy in vivo. Selected inhibitors also showed an antiviral effect against SARS-CoV-2.^17^

Herein we investigated the interaction of (3,5-dichlorophenyl)pyridine-based inhibitors with furin by means of X-ray crystallography as well as biochemical and biophysical assays. Our studies revealed a unique induced-fit binding mechanism of these compounds characterized by major structural rearrangements of furin’s substrate-binding cleft accompanied by slow off-rate binding kinetics.

## Results and Discussion

### Kinetic Characterization of Inhibitor Binding

**Figure 2:**
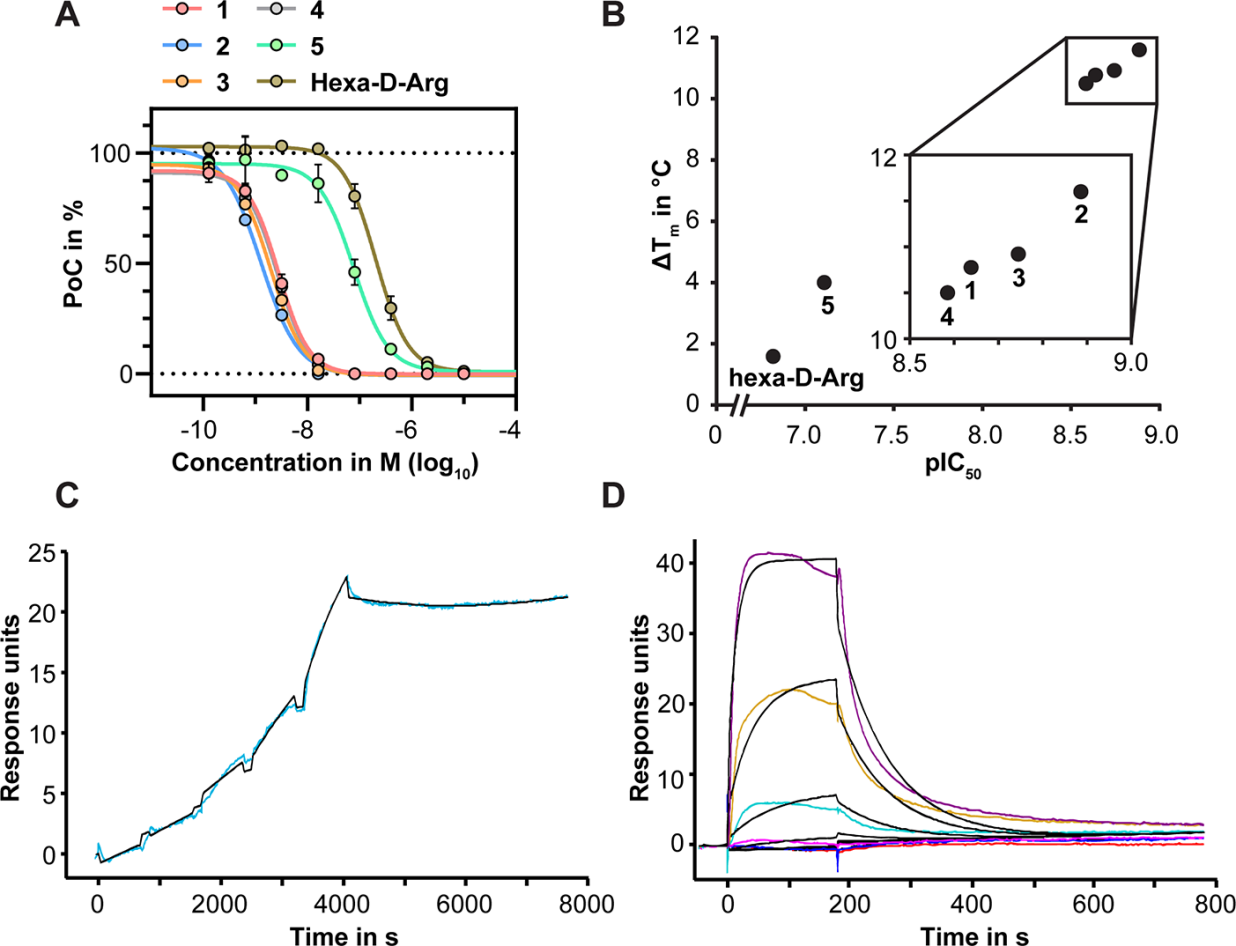
Biochemical characterization of (3,5-dichlorophenyl)pyridine-based furin inhibitors. (A) Representative dose–response curves for **1**–**5** and hexa-d-Arg determined by the MALDI-TOF-MS-based furin activity assay using the TGFβ-derived substrate. (B) Gain of thermostability as a function of pIC_50_ as observed by MALDI-TOF-MS-based potency analyses. The inset shows a magnification for **1**–**4.** (C, D) Surface plasmon resonance (SPR) binding studies of (C) **1** and (D) hexa-d-Arg with immobilized furin. Colored lines represent experimental data, and black lines represent curve fits. The sensograms show representative SPR experiments (single-cycle kinetic for **1** and multicycle kinetic for hexa-d-Arg).

To assay furin’s endopeptidase activity, we established a direct label-free mass spectrometry (MS)-based quantification of the enzymatic product.^18^ For this purpose we employed our recently reported matrix-assisted laser desorption/ionization time-of-flight (MALDI-TOF) MS platform combining label-free detection with high-throughput compatibility.^19,20^ This highly sensitive assay facilitated the measurement of IC_50_ values of tight-binding ligands at very low enzyme concentrations (0.02 nM furin). We tested two peptide substrates, one derived from TGFβ and one from the SARS-CoV-2 S protein, both of which are well-described furin targets (see refs (6) and (21) and Figure S1A). Linear relationships between time and product formation were observed up to 70 and 110 min for TGFβ- and S-protein-derived substrates, respectively (Figure S1B). Next, we investigated the assay-specific substrate concentrations required for half-maximal enzyme velocity. Similar *K*_m,app_ values were observed for the investigated substrates (1.8 and 3.9 μM for TGFβ and S protein, respectively; see Figure S1C), in line with literature data.^21^ As a proof of concept of our assay, we tested the commercially available furin inhibitor hexa-d-arginine and demonstrated good agreement with the literature-described potency (IC_50_: in-house = 152 ± 77 nM (TGFβ)/126 nM (S protein) vs literature = 106 nM^22^). Subsequently, we determined IC_50_ values for the (3,5-dichlorophenyl)pyridine-based furin inhibitors (Figure 2A). Using the TGFβ-derived substrate, we measured IC_50_ values of 2.3, 1.3, 1.8, 2.6, and 78 nM for compounds **1**–**5**, respectively. Using the S-protein-derived substrate, we observed IC_50_ values of 1.1 and 0.8 nM for compounds **1** and **2**, respectively. The results are listed in Table S1 and demonstrate good agreement with the potencies described in the literature.^16^ Similar deviations were observed in other studies with this inhibitor type depending on the substrates used.^17^ Prospectively, the established MALDI-TOF-MS-based activity assay provides the accessibility of high-throughput screening campaigns, enabling the identification of novel chemical starting points for the development of furin inhibitors.

To determine the inhibition mode for compound **1**, we performed enzyme kinetic assays with the fluorogenic PC substrate pyr-ERTKR-7-amino-4-methylcoumarin (pERTKR-AMC). A Dixon plot (*v*^–1^ vs concentration of **1** for substrate concentrations between 4.4 and 13.3 μM; Figure S2A) revealed cross sections of the straight lines in the upper left quadrant, thus indicating a competitive inhibition mode (*K*_i_ = 3.8 ± 0.8 nM; Figure S2B,C).

Interactions of inhibitors with furin increased the structural stability of the protease–inhibitor complexes compared with the ligand-free protease, which was indicated by an increase in the melting temperature (*T*_m_).^23^ We investigated the impact of the (3,5-dichlorophenyl)pyridine-based compounds on the structural stability of furin using differential scanning fluorimetry (nanoDSF^24^). Binding of the inhibitors increased the *T*_m_ of unliganded furin (57.7 ± 0.1 °C) by up to 11.6 °C, as observed for compound **2** (Table S2). The gain in *T*_m_ correlated well with the measured pIC_50_ (i.e., −log_10_(IC_50_)) values (Figure 2B), as reported for substrate-like inhibitors.^10,25^

Next, we investigated the binding kinetics of **1**, **3**, and hexa-d-Arg (control) to immobilized furin in surface plasmon resonance (SPR) experiments (Figure 2C,D and Table S3). As the (3,5-dichlorophenyl)pyridine derivatives bind tightly to furin, we used single-cycle kinetic experiments to determine their off-rates.^18^ The dissociation of **1** and **3** from furin was slow (*k*_off_ = (1.8 ± 0.6) × 10^–4^ s^–1^ and residence time τ = 92 min for **1**; *k*_off_ = (3.1 ± 2.2) × 10^–4^ s^–1^ and τ = 53.7 min for **3**; Figure 2C and Table S3). In contrast, hexa-d-Arg dissociated rapidly from furin and exhibited more transient binding with a fast off-rate. Thus, it was not possible to quantify the off-rate of hexa-d-Arg because of the poor fit of the data (Figure 2D). The on-rates of **1** (*k*_on_ = 4600 ± 2500 M^–1^ s^–1^) and **3** (*k*_on_ = (9.6 ± 2.6) × 10^4^ M^–1^ s^–1^) can be considered as slow and are likely to be influenced by a structural rearrangement needed for binding. Similar behavior showing a slow on-rate was observed for the reversible covalent inhibitor vildagliptin (on-rate to DPP-4 = 7.1 × 10^4^ M^–1^ s^–1^).^26^

### (3,5-Dichlorophenyl)pyridine-Based Inhibitors Induce Major Structural Rearrangements of Furin’s Active-Site Cleft

To investigate the binding mechanism of the (3,5-dichlorophenyl)pyridine-based inhibitors, we soaked compounds **1**–**5** into crystals of unliganded furin and solved the X-ray structures of the protease–inhibitor complexes (Table S4). The structures were refined to resolutions between 1.8 and 1.45 Å. Well-defined electron density maps were observed for all five inhibitors (Figure S3).

**Figure 3:**
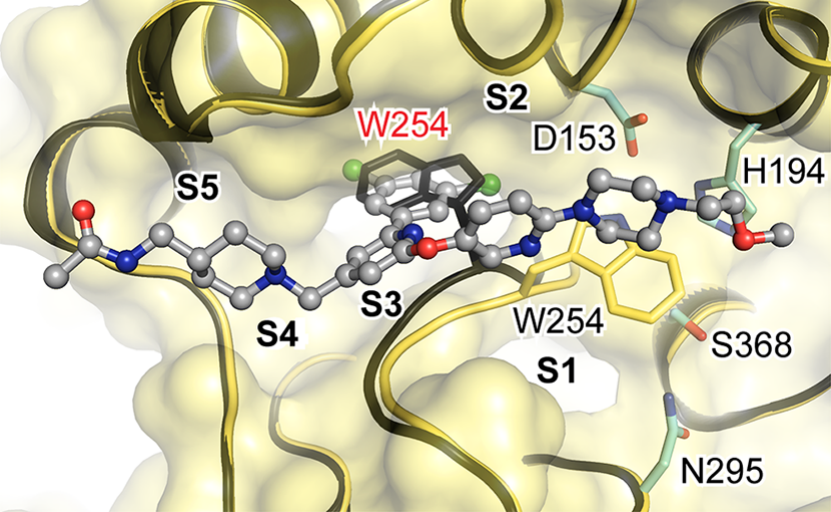
Binding mode of (3,5-dichlorophenyl)pyridine-derived inhibitors. The protease is shown in the cartoon representation (gold) and **1** as a ball-and-stick model (gray). Important residues are shown as stick models (yellow, with catalytic residues in cyan). The transparent molecular surface of furin is superimposed in yellow. The relative locations of the specificity pockets are labeled (S1–S4). Ligand-free furin (PDB ID 5JXG,^5^ black) is superimposed on the furin–inhibitor complex structure. The replacement of Trp254 by the 3,5-dichlorophenyl moiety of **1** should be noted (residues of the unliganded and inhibitor-bound structures are marked in red and black, respectively).

Prototypical conformational changes of the active-site cleft are exemplified by the structure of furin in complex with **1** (Figures 3 and S3A). Upon inhibitor binding, the alignment template (edge strand) moved by 2.1 Å toward Ser368 (based on Cα of Trp254), and the side chain of Trp254 was flipped by approximately 180°. These rearrangements resulted in the formation of an extended hydrophobic surface patch at furin’s substrate-binding cleft (Figure S4A). At the position of the replaced Trp254 side chain, the 3,5-dichlorophenyl moiety inserted into a newly formed hydrophobic binding pocket. Thus, binding of **1** to furin is driven by extensive hydrophobic interactions. Changing the 3,5-dichlorophenyl moiety to a 3-fluoro-5-chloro or 3-fluoro-5-bromo substitution pattern resulted in a 20-fold decrease or 6-fold increase in the potency (examples 11 [inhibitor **4**], 115, and 16 in ref (16)). Substitution of one halogen atom with a bulkier methyl or a difluoromethyl group was also tolerated (examples 61 and 108 in ref (16)), indicating an asymmetry of the hydrophobic binding pocket.

In the inhibitor-bound state, the Trp254 side chain was sandwiched between the (piperazin-1-yl)pyrimidine segment of **1** and the active site. The structural changes of the alignment template also blocked the canonical binding to furin’s S1 binding pocket. Consequently, productive substrate binding to the hydrophobic active-site conformation is not possible. The surface topology and chemistry of the substrate-binding cleft in complex with **1** largely differed from those of unliganded furin and furin bound with a substrate-like inhibitor (compare Figure S4A with Figure S4B,C). Several polar and charged residues are usually involved in canonical protease–inhibitor interactions. At the S1 and S2 pockets, these residues were shielded by the rearranged Trp254 side chain induced by binding of **1**. These conformational changes are in excellent agreement with the slow tight-binding kinetics we observed in the SPR experiments and support an induced-fit inhibition mechanism.

**Figure 4:**
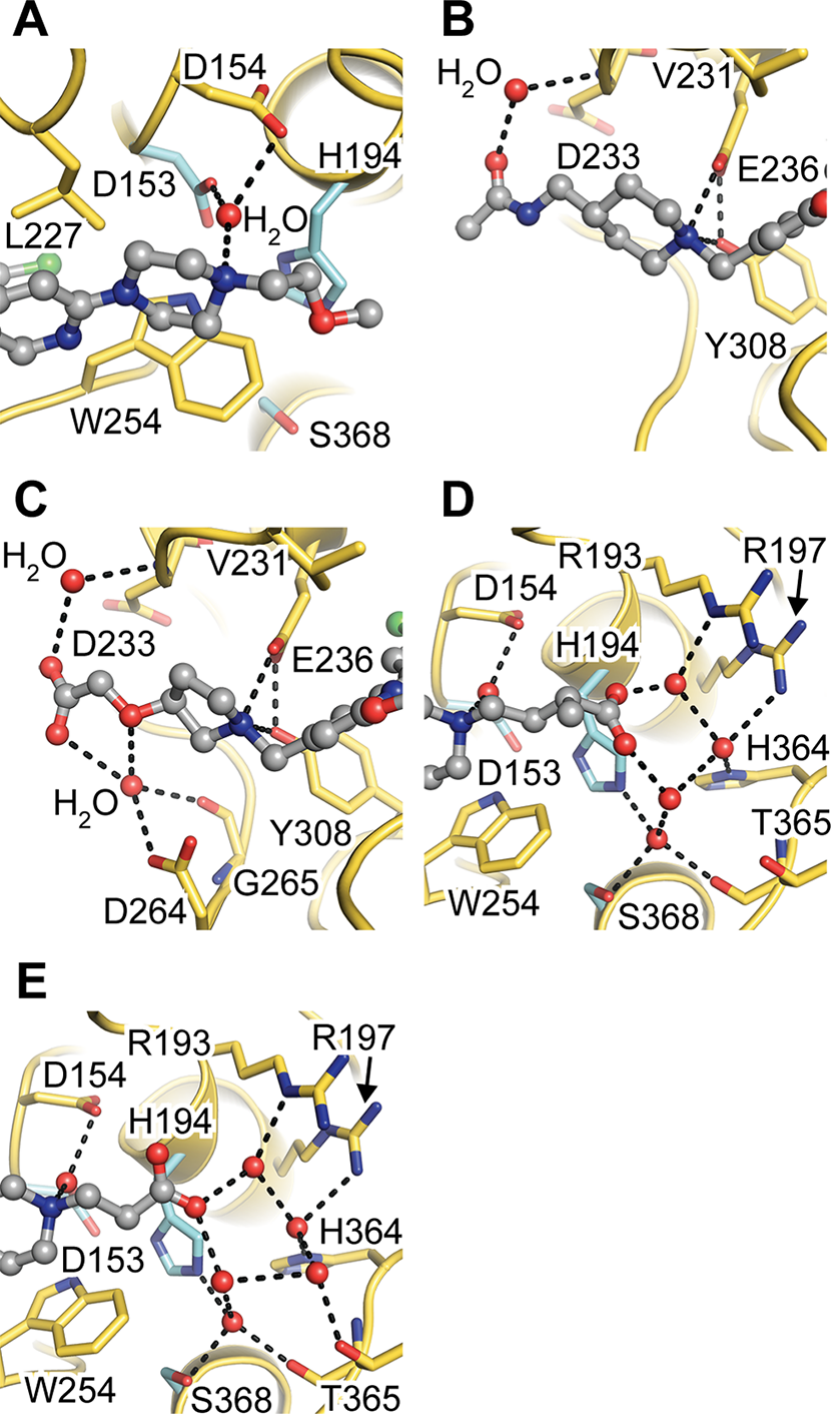
Interactions of (3,5-dichlorophenyl)pyridine-derived inhibitors with the active-site cleft of furin. The protease is shown in the cartoon representation (gold) and the inhibitors as ball-and-stick models (gray). Important residues are shown as stick models (yellow, with catalytic residues in cyan). Water molecules involved in inhibitor–enzyme interactions are shown as red spheres. Important interactions between the inhibitor and furin are marked with dashed lines. (A) Water-bridged electrostatic interaction of the catalytic Asp153 with the piperazine moiety of **1** close to the S1 pocket of furin. (B) Interactions of **1** at the S4/S5 pocket and at the rim of the substrate-binding cleft. (C) Interactions of **5** at the S4/S5 pocket and at the rim of the substrate-binding cleft. (D) Water-bridged interactions of the terminal carboxyl group of **2** close to the S1/S1′ pocket of furin. (E) Water-bridged interactions of the terminal carboxyl group of **4** close to the S1/S1′ pocket of furin.

Inhibitor **1** carries two positive charges that favor electrostatic interactions with furin’s negatively charged substrate-binding cleft. On the basis of the calculated p*K*_a_ of 7.5, N4 of the piperazine ring is expected to be protonated at the pH of the crystals (5.5). Interestingly, we found an indirect interaction by a water-mediated contact with the side chains of Asp153 and Asp154 (Figure 4A). Removal of the piperazine substituent and loss of this contact resulted in a drop in potency (IC_50_ = 20 nM; example 42 in ref (16)) but did not abolish binding. The piperidine ring of **1** should also be protonated and thus positively charged (calculated p*K*_a_ of 8.2; Figure S5). Consistent with this, a salt bridge was found between the piperidine nitrogen and the side chain of Glu236 (Figure 4B). Glu236 is part of furin’s S4 pocket and typically forms a salt bridge with the side chain of P4-arginine of substrate-like inhibitors.

The acetamide motif also interacts indirectly through water-mediated hydrogen bonds with the carbonyl oxygen of Asp233 at the rim of the S4/S5 pocket (Figure 4B).

The displacement of Trp254 by the dichlorophenyl moiety and the interactions of the piperazine and piperidine as described for **1** were also observed for inhibitors **2**–**4**, resulting in very similar binding poses (Figure S6A–C). Accordingly, superpositions of the furin–inhibitor complexes with **1** and **2**, **1** and **3**, and **1** and **4** revealed very similar Cα root-mean-square deviation (RMSD) values of 0.04, 0.05, and 0.08 Å, respectively. In compound **5** the piperidine ring is substituted by a pyrrolidine ring. The pyrrolidine nitrogen of **5** adopts an almost identical position as the piperidine nitrogen of **1** (Figure S6D), maintaining the salt bridge with Glu236 (Figure 4C). A superposition of the furin–inhibitor complexes of **1** and **5** revealed a highly similar overall RMSD value of 0.13 Å. This salt bridge seems to be a major binding element of the (3,5-dichlorophenyl)pyridine-based inhibitors. Interestingly, replacement of the whole piperidine branch of inhibitor **4** by an *N*-methylaminomethyl group (secondary amine) was tolerated (IC_50_ = 7.9 nM; example 50 in ref (16)). Thus, the minimal pharmacophore of the (3,5-dichlorophenyl)pyridine-based inhibitors might include at least a central aromatic anchor (e.g., pyridine), a dihalogenated phenyl substituent, a basic nitrogen that targets Glu236, and a rigid hydrophobic branch that retains the replaced Trp254 (in agreement with the claim of the patent^16^). Nonetheless, the substitution pattern crucially influences the pharmacological properties of these inhibitors. Inhibitors **2**, **3**, and **4** contain negatively charged 2-methylbutanoic or propanoic acid substituents on the piperazine (Figure 1). Enzyme kinetics revealed similar (Table S1) or even higher potency of these inhibitors (Figure 1) compared with **1**. This observation is remarkable on a first glance considering the highly negative net charge of furin’s substrate-binding cleft. Interestingly, we found a well-defined water network that mediates interactions between the carboxylate groups of the inhibitors and positively charged residues of furin’s S1′ region (Figure 4D,E). Both carboxylate oxygen atoms of **2** are bound to a water molecule. In contrast, for **3** and **4** one carboxylate oxygen interacts with two water molecules, adopting a favorable planar binding geometry.

Compound **5** contains only a methyl group at the piperazine ring (Figure 1). On the other terminal end, a negatively charged oxyacetic acid substituent is attached to the pyrrolidine ring. The carboxylate group forms water-mediated hydrogen bonds to Asp233, as observed for the acetamide substituent of the piperidine-containing inhibitors (**1**–**4**). The carboxylate group and the ether oxygen of **5** form a water-mediated contact with the side chain of Asp264 and the carbonyl oxygen of Gly265 (Figure 4C). The negatively charged oxyacetic acid moiety of **5** should be less preferred compared with the acetamide of **1**–**4** at the highly negatively charged S4/S5 region. Electrostatic repulsion of the oxyacetic acid substituent and a better fit of the piperidine ring to the S4/S5 region might explain the reduced potency of **5** compared with **1**–**4**.

The substitution pattern of the (3,5-dichlorophenyl)pyridine-based inhibitors crucially influences their potency and their pharmacological properties. A comparably high bioavailability of **2**, **3**, and **4** was reported in a bleomycin-induced lung fibrosis mouse model.^16^ Total TGFβ production in the lung was reduced by 75%, 86%, and 69% for **2**, **3**, and **4** at 10 mg/kg of body weight, respectively. Compound **2** was orally available in mice, whereas **3** and **4** were injected intraperitoneally.

Importantly, this compound class might also facilitate more selective inhibition of specific PC family members.^17^ Interestingly, the hydrophobic 3,5-dichlorophenyl binding pocket is not conserved among the human PCs (Figure S7). The highest sequence divergence is found for PC7, in agreement with the specificity profile of the compounds.^17^ However, also smaller differences such as those between furin and PC5 might change the steric properties of this binding pocket and thus facilitate PC-specific inhibition. Further optimization of (3,5-dichlorophenyl)pyridine-derived inhibitors could improve furin-specific targeting and hence improve the therapeutic index of these compounds.

During revision of this Letter, another article^27^ reported a structure of furin in complex with a (3,5-dichlorophenyl)pyridine-based inhibitor (BOS318, equivalent to example 207 in ref (16); PDB ID 7LCU, Figure S8A). Superposition of the furin–inhibitor complexes of **1** and BOS318 revealed a similar binding pose (Figure S8B) and a similar RMSD value of 0.20 Å.

## Methods

Compounds **1**–**5** were synthesized as described previously^16^ (**1**–**5** correspond to examples 250, 369, 263, 11, and 128 in ref (16)). For **2** and **5**, racemic mixtures were obtained.

Furin was expressed, purified, and crystallized as described previously.^5,25,15^ On the basis of the fit to the electron density map, the *R* and *S* enantiomers of **2** and **5**, respectively, were modeled in the structures.

Details about the X-ray crystallographic work, the MALDI-TOF-MS-based activity assay, SPR measurements, and thermostability measurements by nanoDSF are available in the Supporting Information.

Structures of the inhibitors **1**–**5** are available in the Protein Data Bank (PDB) under IDs 7QY0, 7QY2, 7QXY, 7QY1, and 7QXZ.
