# Supplementary material for: Dichlorophenylpyridine-Based Molecules Inhibit Furin through an Induced-Fit Mechanism
Source: ACS Chem Biol. 2022 Apr 4;17(4):816–21. doi: 10.1021/acschembio.2c00103 (PMC9016704; doi:10.1021/acschembio.2c00103)
Supplement: Supplementary file 1 — cb2c00103_si_001.pdf [file cb2c00103_si_001.pdf]

Supporting Information

Dichlorophenylpyridine-Based Molecules Inhibit Furin through an Induced-Fit Mechanism.

Sven O. Dahms[1,*], Gisela Schnapp[2], Martin Winter[3], Frank H. Büttner[3], Marco Schlepütz[4], Christian Gnamm[2], Alexander Pautsch[2] and Hans Brandstetter[1]

[1] Department of Biosciences and Medical Biology, University of Salzburg, Hellbrunner Straße 34, A-5020 Salzburg, Austria

[2] Department of Medicinal Chemistry, Boehringer Ingelheim Pharma GmbH& Co KG, 88397 Biberach an der Riß, Germany

[3] Department of Drug Discovery Sciences, Boehringer Ingelheim Pharma GmbH& Co KG, 88397 Biberach an der Riß, Germany

[4] Department of I&R Research, R&D Project Management and Development Strategies, Boehringer Ingelheim Pharma GmbH& Co KG, 88397 Biberach an der Riß, Germany

* Corresponding author e-mail address: sven.dahms@sbg.ac.at

Table of contents

	Page
Supporting Methods	S3-S6
Supporting Figures	
Figure S1	S7
Figure S2	S8
Figure S3	S9-10
Figure S4	S11
Figure S5	S12
Figure S6	S13-14
Figure S7	S15
Figure S8	S16
Supporting Tables	
Table S1	S17
Table S2	S18
Table S3	S19
Table S4	S20
Supporting References	S21

Supporting Methods

X-ray crystallography. Furin was expressed, purified and crystallized as described previously. [1, 2] Soaking was either performed in 1.0 M NaCl, 200 mM Mes/NaOH, pH 5.5, 10% (w/v) PEG8000 and 20% (v/v) DMSO [2] or in 3.13 M NaCl; 100 mM Mes/NaOH; pH 5.5; 200 mM NaH_2PO_4; 1 mM $CaCl_2$ and 20% (v/v) DMSO [3]. Inhibitors **1**, (rac)-**2**, **3**, **4** and (rac)-**5** were soaked at 4 mM, 5 mM, 2 mM 5 mM and 2 mM respectively. Soaked crystals were flash cooled in liquid N_2. Diffraction data collection was performed at the synchrotron beam line BL14.2 [4] (BESSY-II) of the Helmholtz-Zentrum Berlin (HZB) and at beamline X10SA of the Swiss Light source (SLS). The data were processed using XDS [5] with XDS-APP [6] (v2.9) and programs of the CCP4 program suite [7] (v.7.1.001). COOT [8] (v.0.8.9.2) was used for model building. Refinement was performed in PHENIX [9] (v1.18.2, structures of **1**, **2** and **4**) or BUSTER [10] (v2.11.7, structures of **3** and **5**) using the PDB-ID 5JXG [11] as initial model. Geometry restraints of the inhibitors were obtained from the PRODRG-server [12] and with GRADE/MOGUL (http://grade.globalphasing.org). Based on the fit to the electron density map the R- and S-enantiomers of inhibitors **2** and **5** were modelled, respectively. Electron density omit maps were calculated in PHENIX [9] (v1.18.2). PYMOL was used for molecular graphics (http://www.pymol.org) and structural alignments.

MALDI-TOF activity assay. Furin-dependent cleavage of two peptide substrate surrogates, TGFβ (AQHLQSSRHRRALDTNY, *m/z* 2,054.046) and SARS-CoV-2 S protein (TQTNSPRRARSVASQSIIAYT, *m/z* 2,308.219) were monitored via MALDI-TOF-MS. For this purpose, we determined the ratio of formed product peptide (TGFβ: AQHLQSSRHRR, *m/z* 1,375.741, S Protein: TQTNSPRRAR, m/z 1,186.640) to the stable isotope labeled internal standard (TGFβ: AQHLQSSRHR(R), *m/z* 1,385.750, S Protein: TQTNSPRRA(R), m/z 1,196.648)) with an incorporated $^{13}C_6$,$^{15}N_4$-arginine at the indicated position. Enzymatic reactions were set up in assay buffer containing 100 mM HEPES pH 7.5, 1 mM $CaCl_2$, 0.5 mM TCEP, 0.01% (w/v) BSA, and 0.001% (v/v) Tween20. First, 50 nL test compound or DMSO were placed into the wells of a 384-well assay plate. For dose-response experiments, 8-fold dilution series of compound solutions were prepared in DMSO in 1:5 dilution steps starting from 1 mM stock solutions, respectively. Next, 2.5 µL of 2x concentrated furin in assay buffer (0.02 nM final concentration, columns 1-23) or plain assay buffer (columns 24) were added. The plates were then incubated for 10 min in a humidified incubator at 24 °C. Subsequently, 2.5 µL of TGFβ (final concentration 2.5 µM) or S protein (final concentration 5 µM) substrate peptide was added to each well. The plates were mixed for 30 seconds at 1000 rpm and

subsequently incubated for 0.5 h in a humidified incubator at 24°C. After incubation, the enzymatic reaction was stopped by adding 1 µL of respective internal standard (final concentration 1.0 µM) in 3% (v/v) trifluoracetic acid. Dispensing steps were executed with the aid of a Certus Flex Micro Dispenser (Gyger, CH). The plates were then sealed with an adhesive foil, mixed for 30 s at 1,000 rpm and stored at room temperature until preparation of the MALDI target plates. Each 384-well assay plate contained high (no compound; columns 23) and low (no compound & assay buffer instead of enzyme; columns 24) controls to assess compound related activity loss of the enzyme.

MALDI target preparation was performed as described previously with slight modifications.[13] Briefly, a saturated solution of α-cyano-4-hydroxycinnamic acid (HCCA) was prepared in 50% acetonitrile and 50% water (TA50, v/v) containing 10 mM ammonium citrate. The CyBio Well vario liquid handling system (Analytik Jena, GER) equipped with ceramic tips and operated in 384-well format was employed to conduct double-layer spotting providing highly homogeneous spot shapes. Here, 100 nL matrix solution was spotted onto plain steel MALDI target plates and dried in a vacuum chamber. Subsequently, assay plates were centrifuged at 1,000 rpm for 60 s and the seals were removed before 100 nL matrix solution and 100 nL sample were aspirated successively from the matrix reservoir and the assay plate, respectively, and dispensed together onto the dried matrix spots. The MALDI target plate was then dried under vacuum and stored until analysis. Between the first and second spotting step, ceramic tips were washed three times with TA50 containing 10 mM ammonium citrate. After successful transfer of the matrix-analyte-mixture, three repetitive washing cycles using 0.1 M NH_4OH followed by 3 cycles TA50 containing 10 mM ammonium citrate were carried out to prevent carryover and clogging of the tips.

Mass spectra were acquired with a rapifleX MALDI-TOF/TOF instrument from Bruker Daltonics including a Smartbeam 3D laser. FlexControl (v4.0), FlexAnalysis (v4.0), and MALDI PharmaPulse (v2.2) were used for MS-acquisition and data analysis. For the MS-analysis, 5,000 laser shots in 500 shot steps per sample spot were accumulated in positive ionization mode with a 10 kHz laser frequency. The laser power was adjusted manually prior to each measurement to reach a sufficient signal intensity for the internal standard. The acquired spectra were processed with a centroid peak detection set to a signal to noise ratio of S/N = 3 and a Gaussian smoothing (0.02 m/z; 1 cycle). Prior to each campaign, external calibration was performed once with Peptide Calibration Standard II (Bruker Daltonics) containing seven peptides with corresponding $[M+H]^+$ masses: angiotensin II = 1046.5418, angiotensin I = 1296.6848, substance P = 1347.7454, bombesin = 1619.8223, adrenocorticotropic hormone (ACTH) clip 1–17 = 2093.0962, ACTH clip 18–39 = 2465.1983, somatostatin 28 = 3147.10.

Additionally, internal calibration was performed using the monoisotopic peak of the respective internal standard.

MALDI-TOF data, processed with flexAnalysis or MALDI Pharma Pulse, were exported as a comma delimited (.csv) file. Datasets were further processed with either GraphPad Prism (v9.00; GraphPad Software, La Jolla, CA) or in-house laboratory information management system (LIMS) software. Furin activity was tracked by analyzing the measured intensity for the enzymatic product as well as for the corresponding internal standard. Average control values were calculated and set to 100% activity (high controls) and 0% activity (low controls) while the response values of compound-containing wells were normalized against the controls and expressed as percentage of control (PoC). Determination of compound potencies was obtained by fitting the dose-response data to a four-parameter logistical equation.

Enzyme kinetics. Enzyme kinetic measurements were performed in 100 mM HEPES pH 7.5, 1 mM $CaCl_2$, 0.01% (w/v) BSA, 0.001% (v/v) Tween20 with the fluorogenic substrate pyr-ERTKR-7-amino-4-methylcoumarin (pERTKR-AMC, Bachem) and 0.5 nM furin at 24°C. The reactions (100 µl) were initiated by addition of the substrate in 96-well black half area plates (Corning), pre-incubated for 30 min at 24°C and measured for 40 min in a 96-well plate reader (Tecan Infinite 200) at excitation and emission wavelengths of 380 nm and 460 nm, respectively. The K_m value of pERTKR-AMC with furin was determined with 1:2 serial dilution series of the substrate from 100 µM to 0.78 µM. To investigate the inhibition mode of **1** with furin, 1:2 serial inhibitor dilution series were prepared in the concentration range between 100 nM and 0.78 nM and the reaction velocity was determined in presence of 4.4 µM, 8.9 µM and 13.3 µM pERTKR-AMC. Enzyme kinetic data were evaluated with GraphPad Prism. K_m was calculated with the prebuild "Michaelis-Menten" model. For K_i-calculation the data were fitted with a model for competitive inhibition ($v = V_{max}*S/[S+K_m(1+I/ K_i)]$; v = reaction velocity, V_{max} = max. velocity, S = substrate concentration, K_m = Michaelis-Menten constant, K_i = inhibition constant) using a fixed K_m of 9.6 µM (Fig. S2B). The reported K_i-value correspond to the average values observed for the three investigated substrate concentrations. All measurements were performed in triplicates and mean values as well as standard deviations are given.

SPR-experiments. 1, **3** and hexa-D-Arginine [14] were dissolved to a 10 mM stock solution in DMSO. This stock solution was initially diluted 100-fold in running buffer (10 mM HEPES pH 7.5, 100 mM NaCl, 2 mM $CaCl_2$; 0.05% Tween; 1% DMSO) to a concentration of 100 µM and 1% DMSO. The stock solution in 1% DMSO was then further diluted in running buffer with 1% DMSO.

Furin was immobilized onto a NTA-chip after pre-loading with Ni^{2+} using standard procedures. Immobilization levels of furin were in the range of 6000-6500 RU. Binding studies were performed with a Biacore T200 SPR system at 25°C in 10 mM HEPES pH 7.5, 100 mM NaCl, 2 mM $CaCl_2$; 0.05% (v/v) Tween 20, and 1% (v/v) DMSO. Inhibitor **1** and **3** concentrations were 0.24, 0.74, 2.2, 6.6, and 20 nM, and hexa-D-arginine concentrations were 0.16, 0.8, 4, 20, 100, and 500 nM. Binding studies for **1** and **3** were performed in the single-cycle kinetic mode using 700 s association time and 3600 s dissociation time at a flow rate of 30 µl min^{-1}. Binding studies for hexa-D-arginine were performed in the standard kinetic program using 180 s association time and 600 s dissociation time at a flow rate of 30 µL min^{-1}. Sensograms from reference surfaces and blank injections were subtracted from the raw data prior to data analysis, and kinetic parameters were analyzed using the Biacore T200 Evaluation software 3.0 (GE Healthcare). At least three independent SPR experiments were used to calculate the mean values and standard deviation. Residence time τ was calculated as the reciprocal of the mean k_{off} value ($\tau = k_{off}^{-1}$).[15]

Protein thermostability measurement by nanoDSF. The thermostablity of furin-inhibitor complexes was further assessed by nanoDSF.[16] In nanoDSF, protein unfolding was monitored by the change of the intrinsic fluorescence of the protein, mainly from tryptophan and tyrosine residues in the protein. 10 ul 0.2 mg mL^{-1} (3.8 µM) protein solutions (10mM Hepes/NaOH pH 7.5; 100 mM NaCl; 2 mM $CaCl_2$) were loaded into nanoDSF grade standard capillaries (NanoTemper Technologies). The samples were heated up with a ramp rate of 1 °C min^{-1} over a temperature range of 20-95 °C. The thermal unfolding profiles of the proteins were recorded using the Prometheus NT.48 instrument (NanoTemper Technologies). Measurements were performed in duplicate. Unfolding transition temperatures (T_m) were automatically determined by the instrument software.

Supporting Figures

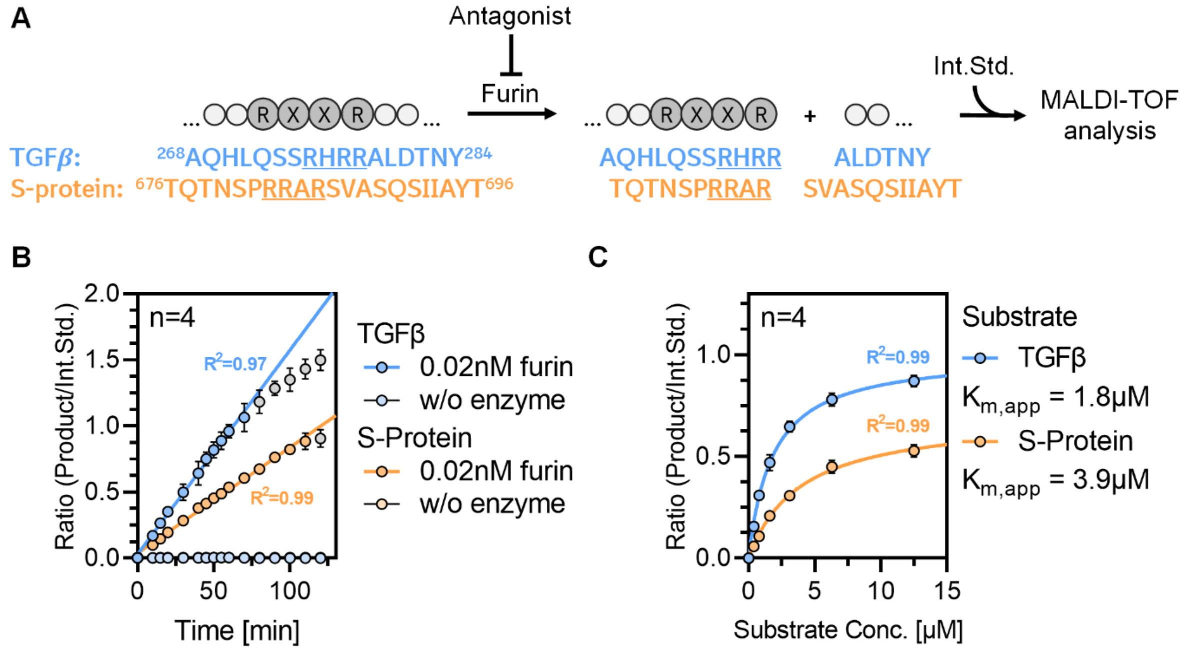

Figure S1 MALDI-TOF-based activity assay. (A-C) Development of a furin *in vitro* activity assay with MALDI-TOF-based readout. (B) Time-dependent linear increase of the enzyme product formation with 2.5 µM TGFβ- (blue) or 5 µM S Protein-derived (orange) peptide substrates in the presence (dark color) or absence (light color) of 0.02 nM furin. (C) K_m determination for TGFβ (blue) or S Protein (orange) peptide substrates. Because the reaction velocities were determined in a stop-point assay, i.e. the reaction velocities are not directly determined as slope from reaction progress curves, the resulting K_m-values are denoted as assay-specific apparent $K_{m,app}$-values.

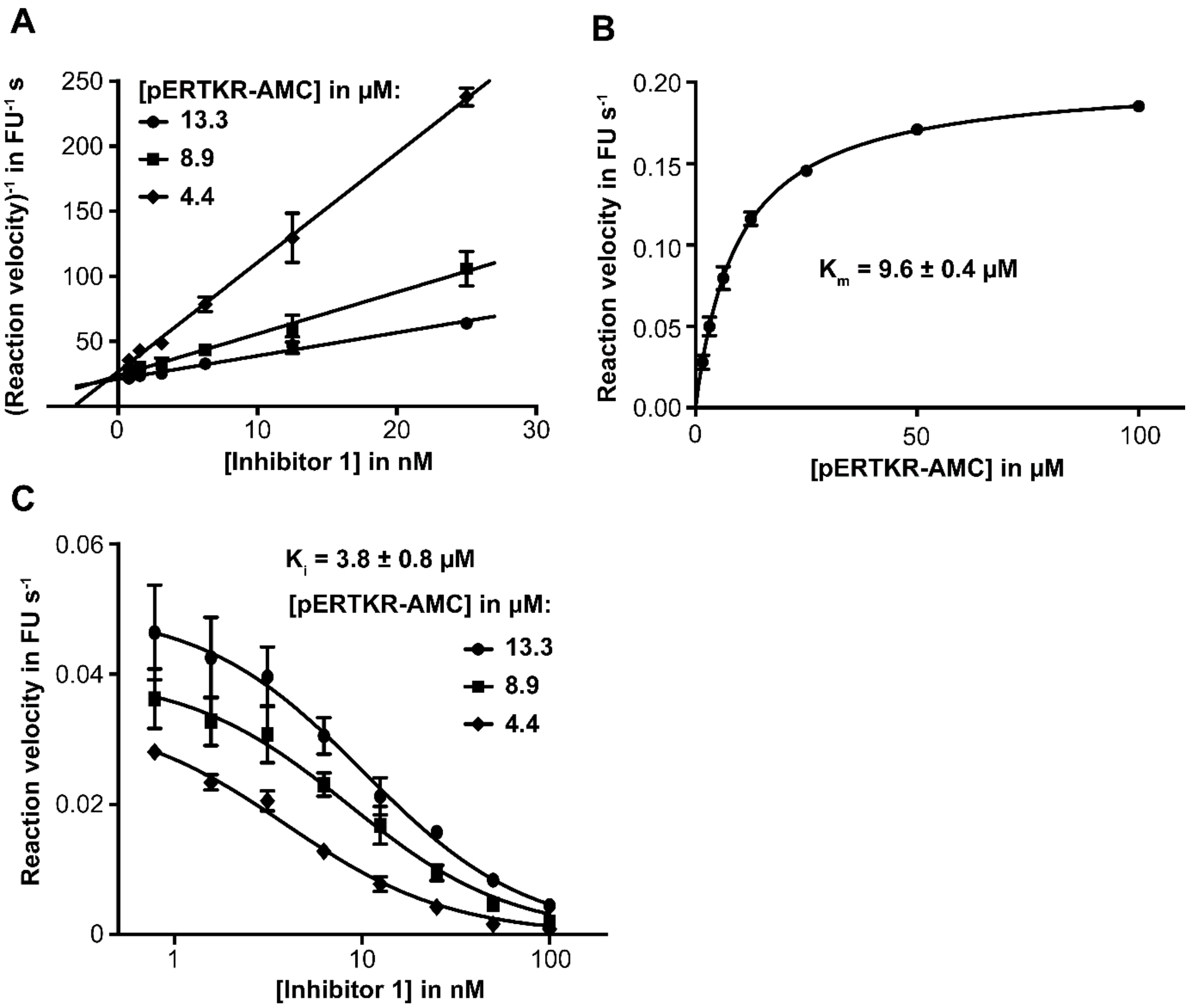

Figure S2 Inhibition mode of inhibitor **1**. (A) Dixon plot of an inhibitor titration series at indicated concentrations of the fluorogenic substrate pyr-ERTKR-7-amino-4-methylcoumarin (pERTKR-AMC). Cross section of the straight lines in the upper left quadrant of the coordinate system indicates a competitive inhibition mode. (B) Michaelis-Menten kinetics of pERTKR-AMC. (C) Non-linear fit of the data with a competitive inhibition model.

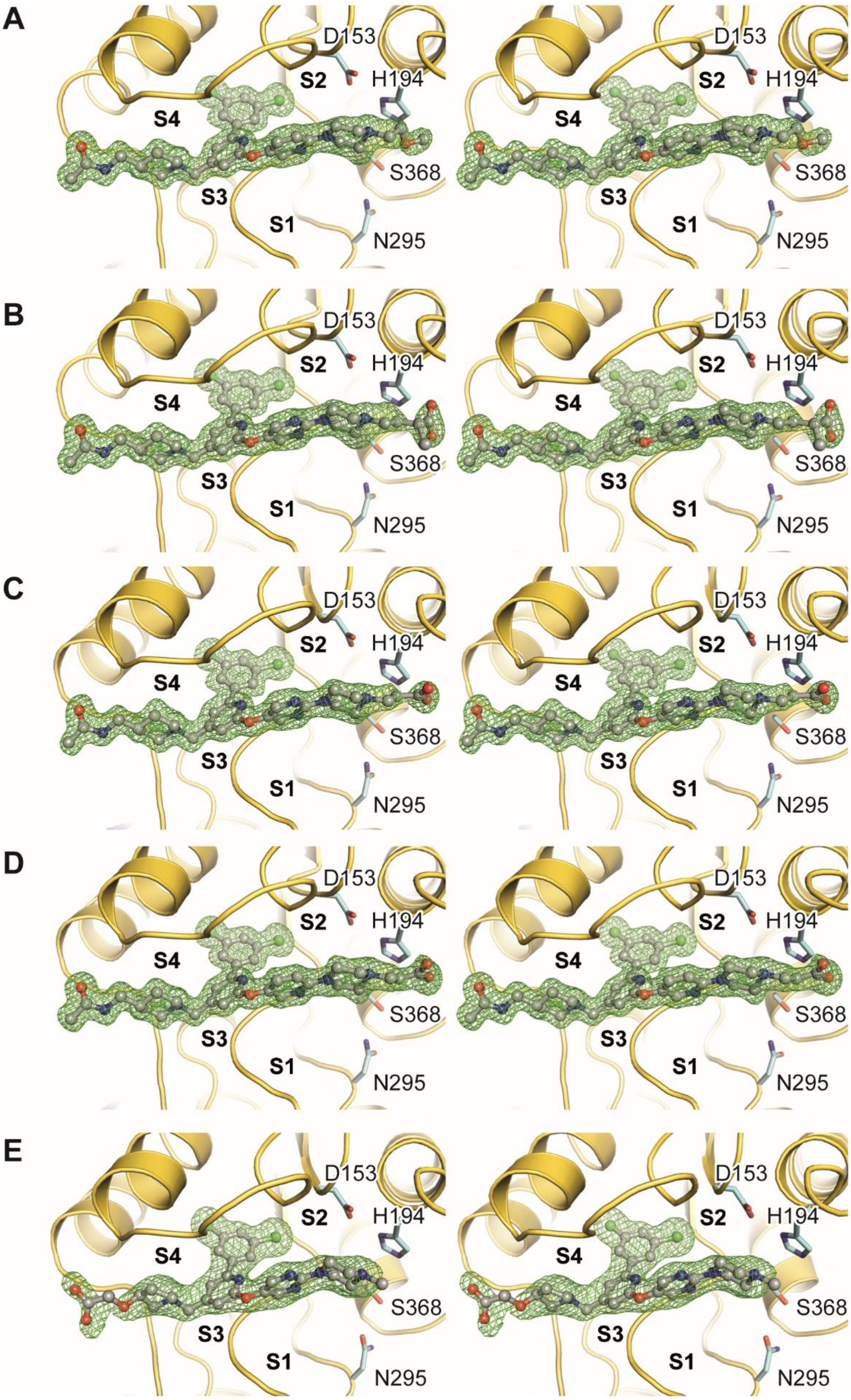

Figure S3 Stereo view of the substrate binding cleft of furin with bound inhibitors **1** (A), **2** (B), **3** (C), **4** (D) and **5** (D). The protease is shown as cartoon representation (gold), catalytic

residues as sticks with carbon atoms in cyan, and the inhibitors as ball-and-stick models, respectively. The $\boldsymbol{F_o}$-$\boldsymbol{F_c}$ annealed omit electron density map of the inhibitors is shown as green mesh and is contoured at 3.0 σ.

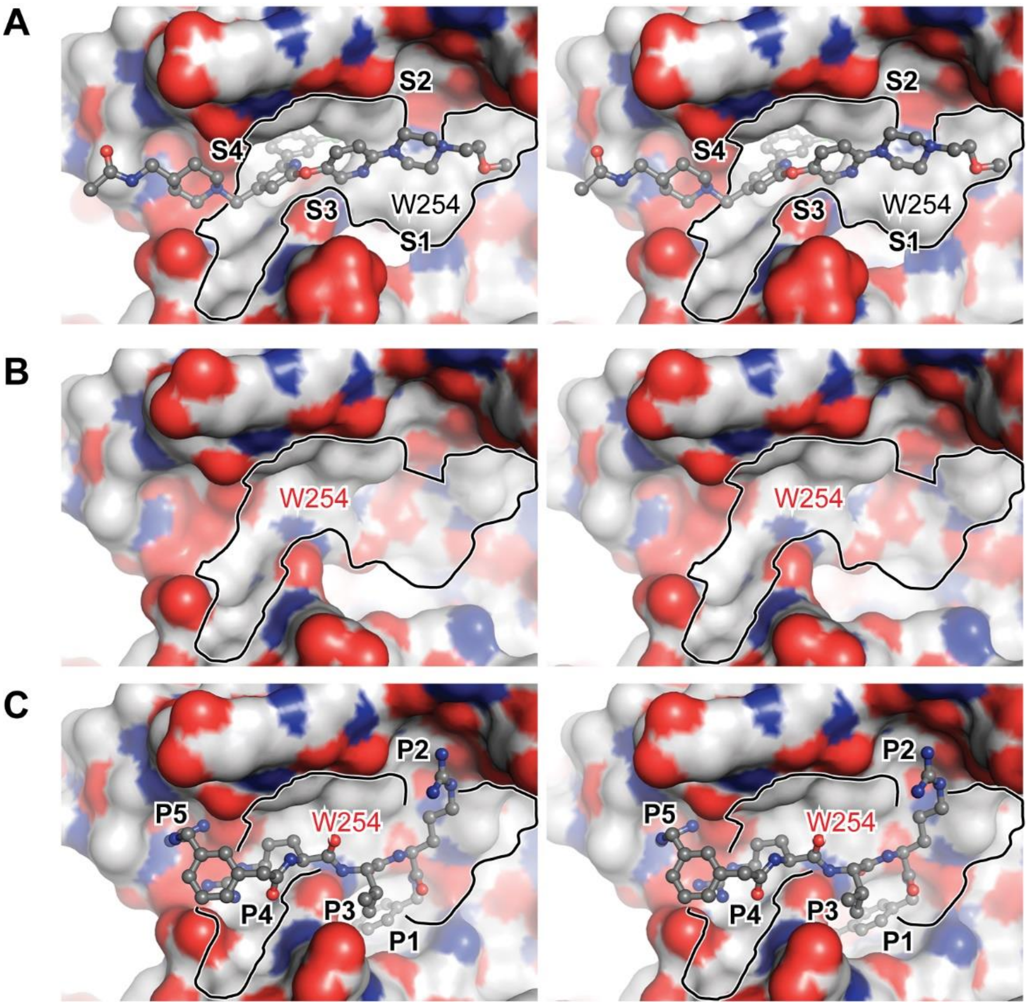

Figure S4 Changes of the molecular surface of furin's substrate binding induced by inhibitor binding. Stereo representations of the molecular surface of furin in complex with (A) **1** (ball-and-stick model, gray), (B) unliganded furin (PDB-ID: 5jxg [11]) and (C) furin bound to the substrate-like inhibitor 3-guanidinomethyl-phenylacetyl-RVR-4-aminomethyl-benzamidine (grey stick model, PDB-ID: 5jxh [11]). Specific furin residues and the inhibitors are shown as stick and ball-and-stick models, respectively. Non-polar surface atoms are colored in gray, polar and charged surface atoms are colored in blue (negative charge or potential) and red (positive charge or potential), respectively. Binding of (3,5-dichlorophenyl)pyridine-derived inhibitors induces a relocation of Trp254 and thus the formation of a large hydrophobic patch at the substrate binding cleft of furin (framed region). Note the changes of the surface properties (shielding of polar and charged residues and remodeling of the shape) induced by binding of **1**.

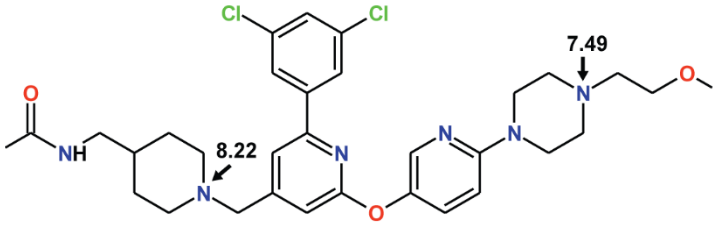

Figure S5 Positively charged nitrogen atoms (marked with arrows) of piperidine and piperazin with calculated pK_a values of **1** as calculated with MoKa.[17]

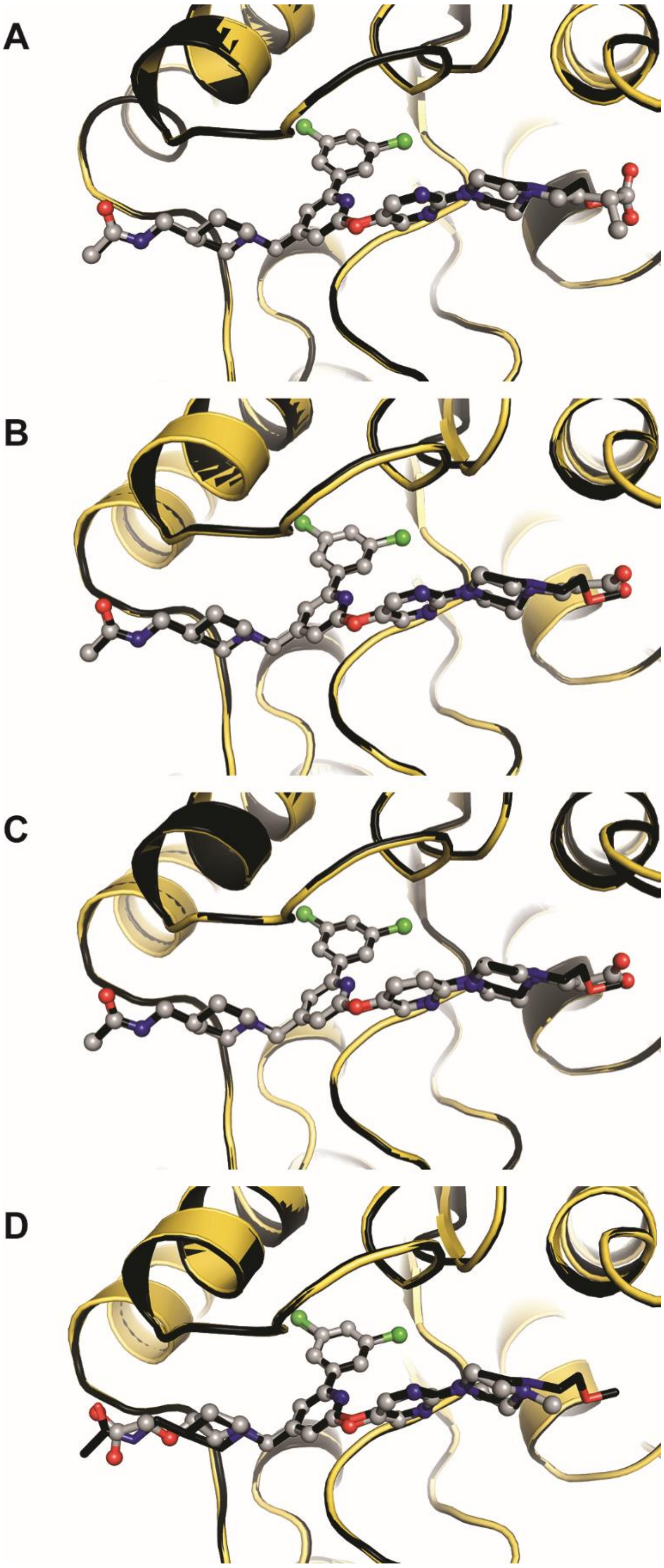

Figure S6 Structural comparison of binding poses of the inhibitors used in this study. The protease is shown as cartoon representation (golden) and **1** is shown as ball-and-stick model

(gray). The structure of furin in complex with **1** (black colored cartoon and stick model) was superposed with the structures of furin in complex with **2** (A), **3** (B), **4** (C) and **5** (D).

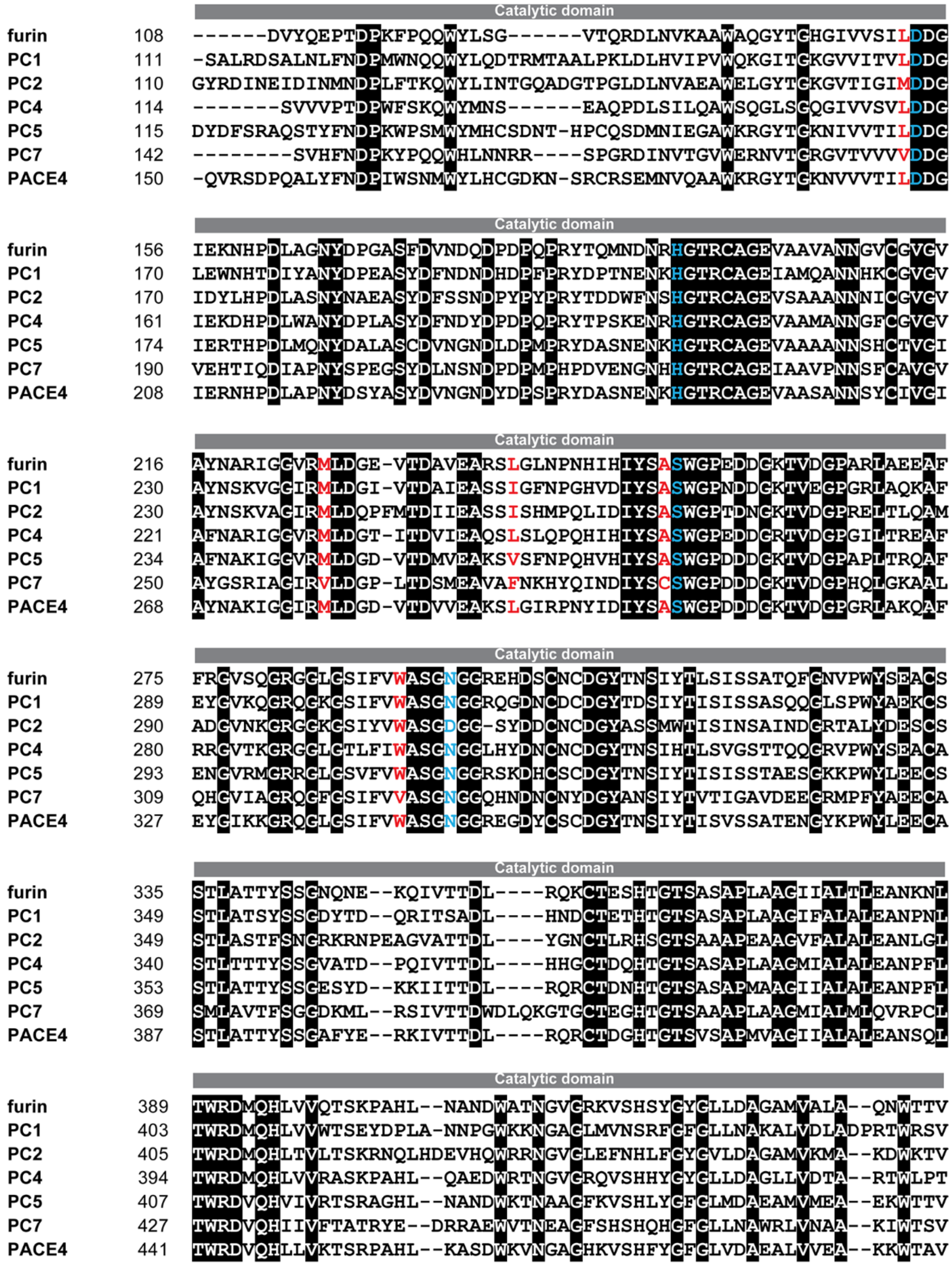

Figure S7 Conservation of the hydrophobic dichlorophenyl-binding pocket. The alignment of the catalytic domains of the human PCs furin (PCSK3), PC1 (PCSK1), PC2 (PCSK2), PC4 (PCSK4), PC5 (PCSK5), PC7 (PCSK7) and PACE4 (PCSK6) was calculated as shown previously.[18] 100% conserved residues are underlined in black. Catalytic residues and residues of the dichlorophenyl-binding pocket are shown in cyan and red, respectively.

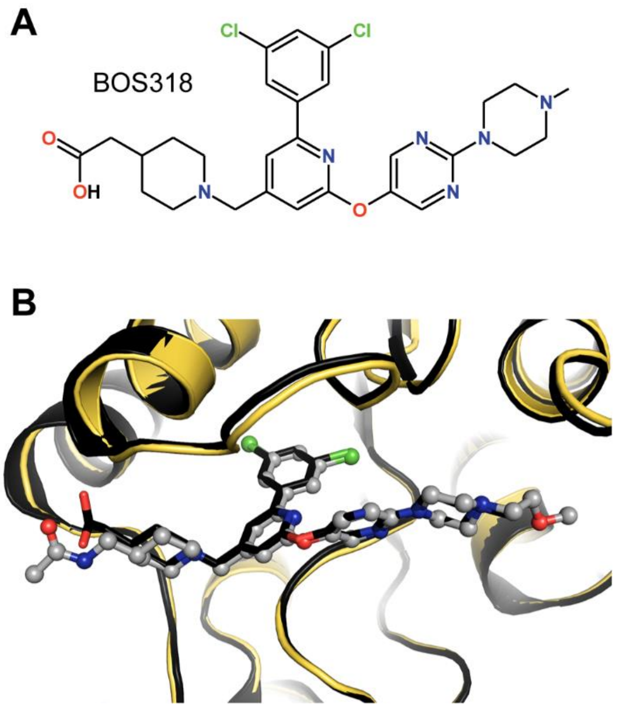

Figure S8 Structural comparison of binding pose of (3,5-dichlorophenyl)pyridine-derived inhibitor with compound BOS318 from PDB-ID 7LCU. (A) Chemical diagram of BOS318. (B) The protease is shown as cartoon representation (golden) and **1** is shown as ball-and-stick model (gray). The structure of furin in complex with **1** was superimposed with PDB-ID 7LCU (black colored cartoon and stick model).

Supporting Tables

Table S1 Compound potencies determined via MALDI-TOF-based enzyme activity assay.

**TGFβ substrate**				
**Inhibitor**	**IC_{50} [nM]**			**IC_{50} [nM]**
	**#1**	**#2**	**#3**	**Mean ± SD[1]**
**1**	2.6	2.4	2.0	2.3 ± 0.3
**2**	1.3			
**3**	2.0	2.3	1.2	1.8 ± 0.6
**4**	2.6			
**5**	78.0			
**Hexa-D-Arg**	207.0	64.2	184.0	152 ± 77
**SARS-CoV-2 S protein substrate**				
**Inhibitor**	**IC_{50} [nM]**			**IC_{50} [nM]**
	**#1**	**#2**	**#3**	**Mean**
**1**	1.0	1.1		1.1
**3**	0.5	1.0		0.8
**Hexa-D-Arg**	126.0			

1: Standard deviation (SD) is given if applicable

Table S2 Thermostability measurement by nanoDSF

Inhibitor	Replicates				Mean (± SD[1]) in°C	ΔT_m in °C
	#1	#2	#3	#4		
**none** [2]	57.7	57.6	57.7	57.6	57.7 ± 0.1	-
**1**	68.5	68.4	-	-	68.4	10.8
**2**	69.4	69.2	-	-	69.3	11.6
**3**	68.7	68.5	-	-	68.6	10.9
**4**	68.3	68.2	-	-	68.3	10.5
**5**	61.8	61.8	-	-	61.8	4.0
**hexa-D-Arg**	59.3	59.2	-	-	59.3	1.6

1: Standard deviation (SD) is given if applicable; 2: Ligand-free furin

Table S3 SPR-analyses of **1**, **3** and hexa-D-Arg

	Replicates				Mean ± SD
	**#1**	**#2**	**#3**	**#4**	
**Inhibitor 1** (τ is 92 min)					
**k_{on} ([Ms]$^{-1}$)**	4531	3563	8463	1672	4600 ± 2500
**k_{off} (s^{-1})**	2.21 x 10^{-4}	8.78 x 10^{-5}	2.21 x 10^{-4}	2.04 x 10^{-4}	(1.8 ± 0.6) x 10^{-4}
**K_D (nM)**	48.71	24.66	26.06	12.18	28 ± 13
**Inhibitor 3** (τ is 53.7 min)					
**k_{on} ([Ms]$^{-1}$)**	6.07 x 10^4	1.23 x 10^5	1.05 x 10^5	-	(9.6 ± 2.6) x 10^4
**k_{off} (s^{-1})**	1.68 x 10^{-4}	6.21 x 10^{-4}	1.37 x 10^{-4}	-	(3.1 ± 2.2) x 10^{-4}
**K_D (nM)**	2.76	5.07	1.31	-	3.1 ± 1.6
**Hexa-D-Arg** (poor curve fit, see Fig. 2D)					
**K_D (nM)**	436.9	965.9	200.4	-	530±320

Table S4 Data collection and refinement statistics.

**Data collection statistics**	**1**	**2**	**3**	**4**	**5**
PDB ID	7QY0	7QY2	7QXY	7QY1	7QXZ
Beamline	BL14.2, HZB	BL14.2, HZB	X10SA, SLS	BL14.2, HZB	X10SA, SLS
Soaking concentration (mM)	4	5	2	5	2
Wavelength	0.9184	0.9184	0.99992	0.9184	0.99992
Space group	$P6_522$	$P6_522$	$P6_522$	$P6_522$	$P6_522$
Unit cell parameters: a = b (Å), c (Å)	131.5, 155.3	131.4, 155.3	131.7, 155.4	131.2, 155.3	132.8, 155.7
Resolution range [a] (Å)	47.1-1.54 (1.63-1.54)	47.1-1.55 (1.64-1.55)	114.0-1.48 (1.65-1.48) [b]	47.1-1.45 (1.54-1.45)	92.5-1.8 (2.05-1.80) [c]
R_{meas} [a] (%)	20.9 (418.2)	26.6 (552.1)	21.0 (284.5)	20.4 (548.3)	35.0 (270.3)
I/sigI [a]	11.6 (0.7)	11.6 (0.6)	16.4 (1.8)	13.4 (0.6)	13.3 (1.8)
$CC_{1/2}$ (%) [a]	99.9 (32.3)	99.9 / (23.2)	99.7 (63.7)	99.9 (29.8)	99.8 / 71.7
Completeness [a]	99.9 (99.2)	98.1 (96.6)	96.5 (80.0)	98.6 (97.1)	96.3 (79.3)
No. of observations (total/unique)	2284347 / 116862	2251620 / 112264	3437519 / 87795	2740536 / 136831	1691747 / 43879
**Refinement statistics**					
No. of non-hydrogen atoms	4378	4373	4386	4387	4278
Protein / inhibitor / water / other	3809 / 43 / 476 / 50	3806 / 46 / 471 / 50	3673 / 44 / 661 / 8	3806 / 44 / 483 / 54	3665 / 39 / 568 / 6
Rwork/Rfree	14.9/ 17.3	16.5 / 18.8	17.1 / 18.2	15.0 / 17.5	18.2 / 21.7
B-factors ($Å^2$)					
Overall/Wilson plot	26.2 / 28.0	27.2 / 28.0	24.2 / 18.9	25.8 / 25.7	27.6 / 24.7
Protein / inhibitor / water / other	24.8 / 23.4 / 36.1 / 38.6	25.7 / 24.6 / 37.9 / 40.2	24.2 / 18.2 / 45.0 / 24.6	24.2 / 20.2 / 37.1 / 40.3	25.6 / 29.2 / 40.9 / 21.6
RMSD bond length (Å)	0.007	0.006	0.008	0.008	0.008
RMSD bonded B-factors ($Å^2$)	1.5	1.4	2.3	1.8	4.5

[a] Values of the highest resolution shell are given in parentheses; [b] Ellipsoidal cutoff (h,k,l): 1.8 Å, 1.8 Å, 1.5 Å; [c] Ellipsoidal cutoff (h,k,l): 2.3 Å, 2.3 Å, 1.8 Å

Supporting References

(1) Dahms, S. O.; Hardes, K.; Steinmetzer, T.; Than, M. E. X-ray structures of the proprotein convertase furin bound with substrate analog inhibitors reveal substrate specificity determinants beyond the S4 pocket. *Biochemistry* **2018**, *57*, 925-934.
(2) Dahms, S. O.; Haider, T.; Klebe, G.; Steinmetzer, T.; Brandstetter, H. OFF-State-Specific Inhibition of the Proprotein Convertase Furin. *ACS Chem Biol* **2021**, *16*, 1692-1700.
(3) Van Lam van, T.; Ivanova, T.; Hardes, K.; Heindl, M. R.; Morty, R. E.; Bottcher-Friebertshauser, E.; Lindberg, I.; Than, M. E.; Dahms, S. O.; Steinmetzer, T. Design, Synthesis, and Characterization of Macrocyclic Inhibitors of the Proprotein Convertase Furin. *ChemMedChem* **2019**, *14*, 673-685.
(4) Mueller, U.; Darowski, N.; Fuchs, M. R.; Forster, R.; Hellmig, M.; Paithankar, K. S.; Puhringer, S.; Steffien, M.; Zocher, G.; Weiss, M. S. Facilities for macromolecular crystallography at the Helmholtz-Zentrum Berlin. *Journal of synchrotron radiation* **2012**, *19*, 442-449.
(5) Kabsch, W. Xds. *Acta crystallographica. Section D, Biological crystallography* **2010**, *66*, 125-132.
(6) Krug, M.; Weiss, M. S.; Heinemann, U.; Mueller, U. XDSAPP: a graphical user interface for the convenient processing of diffraction data using XDS. *J. Appl. Cryst.* **2012**, *45*, 568-572.
(7) Winn, M. D.; Ballard, C. C.; Cowtan, K. D.; Dodson, E. J.; Emsley, P.; Evans, P. R.; Keegan, R. M.; Krissinel, E. B.; Leslie, A. G.; McCoy, A.; et al. Overview of the CCP4 suite and current developments. *Acta crystallographica. Section D, Biological crystallography* **2011**, *67*, 235-242.
(8) Emsley, P.; Lohkamp, B.; Scott, W. G.; Cowtan, K. Features and development of Coot. *Acta crystallographica. Section D, Biological crystallography* **2010**, *66*, 486-501.
(9) Adams, P. D.; Afonine, P. V.; Bunkoczi, G.; Chen, V. B.; Davis, I. W.; Echols, N.; Headd, J. J.; Hung, L. W.; Kapral, G. J.; Grosse-Kunstleve, R. W.; et al. PHENIX: a comprehensive Python-based system for macromolecular structure solution. *Acta crystallographica. Section D, Biological crystallography* **2010**, *66*, 213-221.
(10) Bricogne, G., Blanc, E., Brandl, M., Flensburg, C., Keller, P., Paciorek, W., Roversi, P., Sharff, A., Smart, O. S., Vonrhein, C., and Womack, T. O. *BUSTER version 2.11.7* Global Phasing Ltd.: Cambridge, United Kingdom, 2017.
(11) Dahms, S. O.; Arciniega, M.; Steinmetzer, T.; Huber, R.; Than, M. E. Structure of the unliganded form of the proprotein convertase furin suggests activation by a substrate-induced mechanism. *Proceedings of the National Academy of Sciences of the United States of America* **2016**, *113*, 11196-11201.
(12) Schüttelkopf, A. W.; van Aalten, D. M. PRODRG: a tool for high-throughput crystallography of protein-ligand complexes. *Acta crystallographica. Section D, Biological crystallography* **2004**, *60*, 1355-1363.
(13) Winter, M.; Bretschneider, T.; Kleiner, C.; Ries, R.; Hehn, J. P.; Redemann, N.; Luippold, A. H.; Bischoff, D.; Büttner, F. H. Establishing MALDI-TOF as Versatile Drug Discovery Readout to Dissect the PTP1B Enzymatic Reaction. *SLAS Discov* **2018**, *23*, 561-573.
(14) Cameron, A.; Appel, J.; Houghten, R. A.; Lindberg, I. Polyarginines are potent furin inhibitors. *The Journal of biological chemistry* **2000**, *275*, 36741-36749.
(15) Copeland, R. A.; Pompliano, D. L.; Meek, T. D. Drug-target residence time and its implications for lead optimization. *Nat Rev Drug Discov* **2006**, *5*, 730-739.
(16) Alexander, C. G.; Wanner, R.; Johnson, C. M.; Breitsprecher, D.; Winter, G.; Duhr, S.; Baaske, P.; Ferguson, N. Novel microscale approaches for easy, rapid determination of protein stability in academic and commercial settings. *Biochim Biophys Acta* **2014**, *1844*, 2241-2250.
(17) Cruciani, G.; Milletti, F.; Storchi, L.; Sforna, G.; Goracci, L. In silico pKa prediction and ADME profiling. *Chem Biodivers* **2009**, *6* (11), 1812-1821.
(18) Dahms, S. O.; Creemers, J. W.; Schaub, Y.; Bourenkov, G. P.; Zögg, T.; Brandstetter, H.; Than, M. E. The structure of a furin-antibody complex explains non-competitive inhibition by steric exclusion of substrate conformers. *Scientific reports* **2016**, *6*, 34303.
